# Binge drinking and illicit drug use among adolescent students

**DOI:** 10.11606/S1518-8787.2017051006863

**Published:** 2017-08-25

**Authors:** Jakelline Cipriano dos Santos Raposo, Ana Carolina de Queiroz Costa, Paula Andréa de Melo Valença, Patrícia Maria Zarzar, Alcides da Silva Diniz, Viviane Colares, Carolina da Franca

**Affiliations:** I Departamento Acadêmico. Instituto Federal de Educação, Ciência e Tecnologia de Alagoas. Rio Largo, AL, Brasil; II Hospital das Clínicas. Universidade Federal de Pernambuco. Recife, PE, Brasil; III Programa Nacional de Pós-Doutorado. Programa de Mestrado em Hebiatria. Universidade de Pernambuco. Camaragibe, PE, Brasil; IV Departamento de Odontopediatria e Ortodontia. Faculdade de Odontologia. Universidade Federal de Minas Gerais. Belo Horizonte, MG, Brasil; V Departamento de Nutrição. Centro de Ciência da Saúde. Universidade Federal de Pernambuco, Recife, PE, Brasil; VI Faculdade de Odontologia. Universidade de Pernambuco. Recife, PE, Brasil; VII Departamento de Clínica e Odontologia Preventiva. Universidade Federal de Pernambuco. Recife, PE, Brasil

**Keywords:** Adolescent Behavior, Binge Drinking, epidemiology, Alcoholic Beverages, Street Drugs, Risk Factors, Socioeconomic Factors, Surveys and Questionnaires, utilization

## Abstract

**OBJECTIVE:**

To estimate the prevalence of illicit drug use and its association with binge drinking and sociodemographic factors among adolescent students.

**METHODS:**

This is a cross-sectional study with probabilistic conglomerate sampling, involving 1,154 students, aged 13 to 19 years old, from the public school system, in the city of Olinda, State of Pernambuco, Brazil, carried out in 2014. We used the Youth Risk Behavior Survey questionnaire, validated for use with Brazilian adolescents. The Chi-square test (≤ 0.05) and Poisson regression analysis were used to estimate the prevalence ratios, with 95% confidence intervals.

**RESULTS:**

Use in life of illicit drugs was four times more prevalent among students who reported binge drinking (95%CI 3.19–5.45). Being in the age group of 16 to 19 years, being male, and having no religion were also significantly associated with illicit drug use.

**CONCLUSIONS:**

The prevalence of use in life of illicit drugs was higher in this study than in other studies carried out in Brazil and it was strongly associated with binge drinking. This factor was associated with gender, age, and religion.

## RESUMO

**OBJETIVO:**

Estimar a prevalência do uso de drogas ilícitas e sua associação com *binge drinking* e fatores sociodemográficos entre estudantes adolescentes.

**MÉTODOS:**

Estudo transversal com amostra probabilística por conglomerado, envolvendo 1.154 estudantes, de 13 a 19 anos de idade, da rede pública de ensino, no município de Olinda, PE, 2014. Foi utilizado o questionário *Youth Risk Behavior Survey*, validado para uso com adolescentes brasileiros. Para análise dos dados foi utilizado o teste do Qui-quadrado (≤ 0,05) e análise de regressão de Poisson, para estimar razões de prevalência, com intervalos com 95% de confiança.

**RESULTADOS:**

O uso na vida de drogas ilícitas foi quatro vezes mais prevalente entre os estudantes que relataram o *binge drinking* (IC95% 3,19–5,45). Estar na faixa etária de 16 a 19 anos, ser do sexo masculino e não ter religião também foram significativamente associados ao uso de drogas ilícitas.

**CONCLUSÕES:**

A prevalência do uso de drogas ilícitas na vida foi superior a outros estudos realizados no Brasil e esteve fortemente associado ao *binge drinking.* Esse fator mostrou associação com gênero, idade e religião.

## INTRODUCTION

Illicit drugs are substances that, because of their risk to the health of society, are not permitted by legislation to be marketed and consumed. However, it is estimated that six out of every hundred persons in the world have used an illegal drug^5^. Among these drugs, the most commonly used drugs are marijuana, inhalants, and cocaine, which are associated with several health problems, depending on the frequency and amount of use^5^ ^,^ ^11^.

Adolescents are a vulnerable group for drug use since, at this stage, levels of the dopamine neurotransmitter increase, especially in the prefrontal cortex and limbic system^2^. This increase in dopamine is associated with the use of additive drugs and plays an important role in the reward system^2^ ^,^ ^21^. The use of marijuana, cocaine, and inhalants can directly affect the physical, mental, and social development of adolescents, being associated with mental disorders, crime, dependence, suicide, and death^2^ ^,^ ^5^.

Despite being lawful, alcohol consumption can be a “gateway” to illicit drugs^13^. In addition, the associated use of alcohol with illicit drugs may intensify the deleterious effects that these substances have when consumed in isolation^16^. Alcohol can be consumed in various patterns; one of them is particularly harmful, the binge drinking, which corresponds to the intake of five or more doses for men and four doses for women in a short time interval^1^. Binge consumption, especially when associated with illicit drugs, is still a subject little investigated among adolescents in Brazil^1^ ^,^ ^7^ ^,^ ^18^.

In view of the above, the objective of this study was to estimate the prevalence of illicit drug use and its association with binge drinking and sociodemographic factors among high school students.

## METHODS

This is a cross-sectional study with a probabilistic sample of low-income adolescents enrolled in public schools in the city of Olinda, State of Pernambuco, Brazil. Approximately 75% of the high school students of Olinda are enrolled in the 32 state public schools^a^. The municipality of Olinda has 390,000 inhabitants, high human development index (HDI = 0.735) and high demographic density (9,063.58 inhab./km^2^), being the fifth largest in Brazill^a^.

To estimate sample size, we used an estimate of the prevalence (p) of 15% of use in life of illicit drugs, based on data obtained in the pilot study, with a 3% margin of error (d) and a 95% confidence level (z = 1.96). Considering that the sampling design was a conglomerate one, we corrected the design effect (c) in 2.1. Using the sample formula^9^ [n = (z^2^ x p x 1 - p x c) / d^2^], the minimum sample size was 1,143 sample units. In order to correct for any losses, we increased the sample size by 2% [100 / (100 - 2)], amounting to 1,167 students.

The procedure to select the conglomerates was done in two steps. In the first step, we randomly selected 22 schools (69% of all public schools) and, in the second step, we randomly selected the classes. The eligibility criteria for this study were: adolescent students of both genders, aged 13 to 19 years, and regularly enrolled in high school in the state’s public schools of the municipality. We excluded students with a disability that made them unable to self-administer the questionnaire, as well as students with 20% or more of unanswered questions.

Data were collected from April to August 2014 in classrooms without the presence of the teacher. The application time ranged from 30 to 60 minutes, depending on the number of students in the class, which ranged from 20 to 50 students. The objectives of the research were explained in all the rooms, as well as the guarantee of the anonymity of the respondents.

The instrument used was a validated version of the Youth Risk Behavior Survey (YRBS) – Brazil^8^. This questionnaire was developed by the Centers for Disease Control and Prevention (CDC) and presents 10 modules of risk behaviors among adolescents and young persons. For this study, three modules were used: alcohol, with three questions, marijuana, with two questions, and other drugs, with two questions on cocaine and one on inhalants. In addition to this questionnaire, demographic and socioeconomic information was collected to identify gender, age, maternal education, and type of education.

The two researchers and assistants were instructed and trained by a member of the research group to apply the questionnaire, in order to standardize data collection in all classes. A pilot study was carried out in five public schools in the city of Olinda to subsidize data for sample calculation and to evaluate the calibration of the researchers. The reproducibility of the answers obtained in the application of the questionnaire in the pilot study was calculated by modules and ranged from 0.642 to 1.00, and there was no need to change the methods.

The dependent variable was the use in life of illicit drugs, defined as the use of, at least once in life, marijuana, cocaine, or inhalants. The independent variables were divided into demographic (age and gender), socioeconomic (maternal educational), related to school (type of education), and binge drinking, which was defined as the consumption of five or more doses of alcoholic beverages on one same occasion.

The demographic and socioeconomic variables were dichotomized into: age (13–15 and 16–19 years)^17^ and maternal education (≤ 8 and > 8 years of education). Binge drinking was evaluated in the 30 days prior to the research and dichotomized into yes and no^12^. The type of education was considered as *regular* when the schools had classes in only one period, morning or afternoon, as *semi-integral* when it had classes in the two periods, morning and afternoon, on two days of the week, and as *integral* when it had classes in the two periods, morning and afternoon, in the five days of the week.

The data were tabulated in double entry in the software Epidata 3.1, and the errors found in the validation were corrected. In the description of the proportions, the binomial distribution was approximated to the normal distribution by the 95% confidence interval. The Chi-square test (p ≤ 0.05) was used to verify the association between the dependent variable and the independent variables.

Poisson regression was applied in order to estimate the prevalence ratio (PR) between the use in life of illicit drugs (dependent variable) and the independent variables. Among the variables considered as independent, we included those that presented a value of p < 0.20 in the Poisson regression. Data analysis was performed using the statistical program Statistical Package for Social Sciences (SPSS) for Windows, version 21.0 (SPSS Inc, Chicago, IL, USA).

The approval of the study was obtained by the Ethics Committee of the *Universidade de Pernambuco* (CAAE 13800813.7.0000.5207) and is in accordance with the Declaration of Helsinki. All participants signed a consent form and their parents consented passively.

## RESULTS

The final sample corresponded to 1,154 students, which represented a loss of 1.1% from refusals and incomplete questionnaires. A little more than half (53.9%) were female and maternal education was more than eight years (53.3%). Most were enrolled in regular education (65.2%), between 16 and 19 years old (72.3%), and affiliated with some religion (72.7%). The prevalence of illicit drug use (marijuana, inhalants, or cocaine) was 15.8% (95%CI 13.7–18.0), while binge drinking was 23.1% (95%CI 20.5–25.4).

Table 1 lists the participants according to illicit drug use, sociodemographic factors, and type of education. Illicit drug use was not associated with maternal education and type of education.

**Table 1:** Illicit drug use (marijuana, cocaine, or inhalants) according to binge drinking and the sociodemographic and school-related variables among high school students. Olinda, State of Pernambuco, Brazil, 2014. (n = 1,154)

Variable	Illicit drugs				p

	Yes		No		
	
	n	%	n	%	
Gender^a^					0.014
Female	81	13.1	535	86.9	
Male	97	18.4	429	81.6	
Age (years)					< 0.001
13–15	23	7.2	297	92.8	
16–19	159	19.1	675	80.9	
Religion^b^					< 0.001
Yes	114	13.6	725	86.4	
No	68	22.2	238	77.8	
Type of education					0.263
Regular	112	14.9	640	85.1	
Semi-integral/Integral	70	17.4	332	82.6	
Maternal education^c^					0.797
> 8 years	64	15.0	362	85.0	
≤ 8 years	76	15.6	410	84.4	
Binge drinking^d^					< 0.001
Yes	107	39.5	164	60.5	
No	75	8.5	804	91.5	

Missing values ^(a)^ 12 (1%); ^(b)^ 9 (0.7%); ^(c)^ 242 (20.9%); ^(d)^ 4 (0.3%).

Table 2 shows the results of the crude and adjusted Poisson regression analysis for use in life of illicit drugs (marijuana, cocaine, or inhalants) according to the independent variables. Students who reported binge drinking had a 317% higher prevalence of reporting illicit drug use compared to those who did not binge drink. Being in the age group of 16 to 19 years increased the prevalence of illicit drug use by 120% compared to those aged 13 to 15 years. No religious affiliation increased by 37% the prevalence of drug use when compared to students who reported having religion. Being male also increased the prevalence of illicit drug use.

**Table 2:** Crude and adjusted prevalence ratio of the association between illicit drug use and the independent variables of the study. Olinda, State of Pernambuco, Brazil, 2014. (n = 1,130)

Variable	PR (crude)	95%CI	p	PR (adjusted)	95%CI	p
Gender						
Female	1	-	-	1	-	-
Male	1.40	1.07–1.84	0.014	1.33	1.03–1.72	0.028
Age (years)						
13–15	1	-	-	1	-	-
16–19	2.65	1.75–4.03	< 0.001	2.21	1.47–3.31	< 0.001
Religion						
Yes	1	-	-	1	-	-
No	1.64	1.25–2.14	< 0.001	1.37	1.06–1.77	0.016
Binge drinking						
Yes	4.63	3.56–6.01	< 0.001	4.17	3.19–5.45	< 0.001
No	1	-	-	1	-	-

## DISCUSSION

The prevalence of illicit drug use (marijuana, cocaine, or inhalants) was 15.8%, which is higher than that found in other studies in Brazil (2.4% in a city in the South^20^, 6.9% in the State of Pernambuco^4^, and 7.3% in Brazil^10^).

The variable that was most strongly associated with illicit drug use (marijuana, cocaine, or inhalants) among adolescents was binge drinking, similar to the findings of other studies with representative samples of adolescent students which investigated illicit drugs separately^7^ ^,^ ^18^ ^,^ ^19^. A longitudinal study conducted in the United States showed binge drinking as a predictor (OR = 1.91; 95%CI 1.39–2.63) for beginning marijuana use^19^. In Brazil, binge drinking increased the risk of inhalant use (OR = 5.02; 95%CI 2.57–9.81)^18^ and was associated with cocaine and marijuana use (p < 0.001)^7^.

After binge drinking, the factor associated with greater strength was the age of illicit drug use. The onset of illicit drug use usually occurs in intermediate adolescence (13 to 15 years) while the beginning of alcohol use occurs in the initial phase (from 10 to 12 years)^15^ ^,^ ^20^. This partially explains the increased use in life of illicit drugs (marijuana, cocaine, or inhalants) by students in the age group of 16 to 19 years in this study, reinforcing the importance of drug use prevention in early adolescence.

The third factor associated with illicit drugs use was religion. The higher consumption among adolescents who reported having no religion could be considered similar to other studies that identified religion as a protective factor for the use of drugs among adolescent students^3^ ^,^ ^6^, possibly because of the norms of conduct disseminated by most religions.

The higher prevalence (33%) of drug use among male adolescents observed in this study shows that this group is still at higher risk^4^ ^,^ ^10^ ^,^ ^11^; although, studies also indicate some similar risk behaviors between genders^7^ ^,^ ^18^ ^,^ ^20^.

Another factor investigated was type of education. In Brazil, as well as in the municipality investigated in this study, the More Education Program was implemented in integral schools in 2007, which aims to offer various activities for the prevention of risk behaviors, among them use of alcohol, tobacco, and other drugs^14^. However, the students of semi-integral or integral schools did not present significant differences in relation to the students of regular schools. This result is a cause for concern, as it is expected that the longer stay of adolescents in the school environment will protect these students from drug use.

Some research limitations should be considered. One of them is the cutoff point of binge drinking, which may have been underestimated for females, since its measure of consumption should be four doses or more, and we considered five doses or more for both genders in this study. Another limitation was the low variability of socioeconomic data, because although a probabilistic conglomerate sampling was carried out, all respondents were enrolled in public schools, in which most have low economic status. Overcrowding of students per room in some classes (50 students) may also have been a limitation because it may have inhibited the answers or contributed to the homogeneity of responses.

We can conclude that the prevalence of illicit drug use (marijuana, cocaine, and inhalants) was high compared to other studies in Brazil. The habit of binge drinking, being male, being aged between 16 and 19 years, and having no religion were variables that showed an independent association, each with illicit drug use. Binge drinking was the variable that most influenced the use of marijuana, cocaine, and inhalants; however, because this is a cross-sectional study, we cannot affirm its predictive effect on the use of these drugs.

Thus, longitudinal studies that respond to this issue in different socioeconomic and cultural contexts are important, as well as the development, implementation, and monitoring of harm reduction and prevention policies and actions focused on alcohol consumption.

## Footnotes

a Instituto Brasileiro de Geografia e Estatística. Cidades@: Pernambuco - Olinda. Rio de Janeiro: IBGE; 2010 [cited 2017 Apr 30]. Available from: http://cidades.ibge.gov.br/xtras/perfil.php?codmun=260960
